# Panhandle Medical Society. Austin District Medical Society. U. S. Practitioners’ Protective Alliance. Central Texas Medical Society. Circular Letter Texas Texas State Medical Association

**Published:** 1891-02

**Authors:** 


### Society Notes.

Another.—The Panhandle Medical Society was organized at Vernon, on the 24th inst. Particulars in next issue.

Special Attention.—The Austin District Medical Society will hold its next regular quarterly meeting in Austin on the 3rd Thursday, 19th day of March. Delegates to the Waco meeting of the State Association (April 28) will be elected at this meeting. A splendid programme has been prepared. Be sure to attend.

---

The United States Medical Practitioners Protective Alliance is the name of an organization of which Dr. J. H. DeWolff, of Baltimore, is the founder. Dr. W. H. Crim, of ——— is President, and Dr. J. F. Davison, of Glendola, N. J., is Secretary. The admittance fee is $3, and annual dues $2. The object of the association is, "to maintain organized co-operation amongst practicing physicians who are legally qualified to practice in their respective States, etc., for the purpose of protecting practitioners from the abuse of dispensaries that treat free those who are able to pay; from unjust competition caused by short term, quick graduating and inferior medical colleges; to endeavor to promote the passage of just and equitable laws regulating the practice of medicine," etc. Address the Secretary.

---

WACO, TEXAS, January 15, 1891.

The next meeting of the Central Texas Medical Association will take place Tuesday, April 14, 1891, for which the following programme has been appointed:

1. "Puerperal Pelvic Peritonitis."—By Dr. H. C. Ghent; discussion to be opened by Drs. Douglas, Harris and J. M. Frazier.

2. "Anodynes and How to Use Them."—By Dr. S. D. Davison; discussion to be opened by Drs. W. W. Wilkes and J. T. Valliant.

3. "Eczema."—By Dr. N. A. Olive; discussion to be opened by Drs. W. B. Carpenter and J. E. Brown.

4. "Pulmonary Tuberculosis."—By Dr. M. D. Knox; discussion opened by Drs. H. L. Taylor and S. V. Wagner.

5. "Treatment of Fractures of the Long Bones."—By Dr. H. W. Brown; discussion to be opened by Drs. W. C. Blalock and A. M. Curtis.

Volunteer papers and reports of cases are cordially invited.

DANIEL PARKER, President.

W. O. WILKES, Secretary.

The following letter has been issued from the office of the Secretary of the Texas State Medical Association:

TEXAS STATE MEDICAL ASSOCIATION,
EXECUTIVE OFFICE, FORT WORTH, Feb. 8, '91.

*Dear Doctor:* The 23rd annual meeting of our Association wild be held at Waco on the 4th Tuesday (28th day) of April next, and remain in session four days.

Waco, the central city of the State, with its beautiful location, admirable railroad facilities, flowing wells of pure water, and hospitable inhabitants, is a fit place for such a meeting. The city has invited us as its guests, and her known reputation for hospitality is a guarantee that everything possible will be done to make our visit pleasant and, profitalbe. The time of meeting will be the loveliest season of the year.

Many subjects of great importance will be discussed by some of the brightest physicians of Texas. No live members of the profession can afford to be absent. The committee of arrangements will make the best possible rates with railroads and hotels.

We not only earnestly entreat you to give us the pleasure of your association and the benefit of your experience and intellect, but sincerely hope that you will make an extra effort to bring as many regular physicians as possible with you. Let no ordinary obstacle keep you away. We are proud of our organization now, which is respectable and respected for its numbers and intelligence, and we can still greatly improve it by inducing other bright physicians to join it, and by thorough discussion of new themes.

With pleasant anticipations of meeting you at Waco, believe me, Sincerely yours,

*Official:* W. P. BURTS, M. D., President

F. E. DANIEL, M. D., Secretary.

---

C. M. Ramsdell, A. M., M. D., of Lampasas, has been elected President of the Alliance University at Marble Falls. Dr. Ramsdell is a prominent member of the Texas State Medical Association, and ex-chairman of Section on Surgery. While congratulating him upon the honor conferred upon him, we regret his retirement from the practice of a profession to which he is an ornament.

